# Toxic Alcohol Ingestion: A Case Report and Review of Management Pathways

**DOI:** 10.7759/cureus.13092

**Published:** 2021-02-03

**Authors:** Paavani Atluri, Deepa Vasireddy, Srikrishna V Malayala

**Affiliations:** 1 Internal Medicine, Bay Area Hospital, Coos Bay, USA; 2 Pediatrics, Pediatric Group of Acadiana, Lafayette, USA; 3 Internal Medicine, Temple University Hospital, Philadelphia, USA

**Keywords:** toxic alcohol, methanol, ethylene glycol, diethylene glycol, propylene glycol, isopropanol, fomepizole

## Abstract

Toxic alcohol ingestion can be fatal or produce irreversible tissue damage and hence timely recognition and treatment are very important. The physician has to often rely on clinical features and different lab values in order to derive the possible causative agent consumed. Gas chromatography is the definitive diagnostic test to detect the toxic alcohol but is unfortunately not available to run in house in most hospital laboratories in the acute clinical setting. We present a 41-year-old gentleman who was brought to the ED for further evaluation of vomiting and upper abdominal pain. Our clinical impression was that the patient had possible mixed toxic alcohol ingestion. General principles and treatment of alcohol intoxication include gastric lavage or use of activated charcoal. Administration of ethanol or fomepizole to delay or prevent generation of toxic metabolites needs to be initiated while sufficient alcohol remains and metabolized and measurement of blood alcohol concentrations and/or serum osmolality can be helpful. Dialysis is helpful in removing unmetabolized alcohol and possibly toxic metabolites and delivering base to patients to ameliorate metabolic acidosis.

## Introduction

Methanol, ethylene glycol, diethylene glycol, propylene glycol, and isopropanol are the common alcohols whose consumption can lead to toxic ingestions [1]. Toxic ingestions can lead to neurological impairment of the patient and can make eliciting an accurate history of what was consumed challenging for the treating physician [2]. The physician has to often rely on clinical features and different lab values in order to derive the possible causative agent consumed. Gas chromatography is the definitive diagnostic test to detect the toxic alcohol but is unfortunately not available to run in house in most hospital laboratories in the acute clinical setting [3]. According to the 2019 National Survey on Drug Use and Health (NSDUH), 25.8% of people aged 18 or older reported that they engaged in binge drinking in the past month while 6.3% reported that they engaged in heavy alcohol use in the past month [4]. An estimated 95,000 people with a male predominance died every year from alcohol-related causes [4-5]. Alcohol is the third leading preventable cause of death in the United States [4].

## Case presentation

We present a 41-year-old gentleman with a known history of alcoholism who was brought to the ED by emergency medical services for further evaluation of vomiting and upper abdominal pain. The patient usually drinks two to three beers at least two to three times per week and as he ran out of alcohol, he admitted to drinking two shots of industrial alcohol. Interpreter services were used as the patient was Spanish speaking. The patient was alert, awake and oriented in time, place and person. He was very tremulous on admission. He denied having headache, vision changes, chest pain, chest pressure, shortness of breath, abdominal pain, recent changes in bowel or bladder habits. He admitted to mixing rubbing alcohol with soda and the amount of ingestion of industrial alcohol remained unclear. Blood alcohol was checked on admission and was noted to be negative at less than 10 mg/dL. He did have significant anion gap metabolic acidosis with compensated severe metabolic alkalosis and respiratory alkalosis. Usually anion gap is not seen in patients with isopropyl alcohol ingestion and hence upon further investigation the patient is not sure as to what he consumed. 

He was noted to have lactic acidosis of 4.4 mmol/L, severe hypokalemia of 2.6 mmol/L, bicarbonate level of 20 mmol/L, anion gap of 29 mmol/L, beta hydroxybutyrate of 1.75 mmol/L, serum osmolality of 318 mOsm/kg; his acetaminophen level was negative at less than 10 ug/mL, salicylate level was less than 1 mg/dL and hyperbilirubinemia level was at 3.2 mg/dL. Initial venous blood gas showed a pH of 7.81 with a PCO2 of 11 and PO2 of 54. Arterial blood gas (ABG) was obtained to follow-up on the abnormal pH and on ABG pH was 7.63 with PCO2 of 26.6, PO2 of 138 on room air, and bicarb was 28.2 with calculated oxygen saturations of 99%. INR was at 1.1 and pro time was 12.9 s, hemoglobin level 14.1 g/dL, macrocytosis with mean corpuscular volume 106 fL, mean corpuscular hemoglobin 37.9 PG, and thrombocytopenia with platelet count 100,000/uL. White blood cell count was noted to be normal. Urinalysis was positive for 2+ proteinuria, 2+ glucosuria, and 3+ ketonuria. 

Urine toxicology screen was positive for tetrahydrocannabinol. Two sets of blood cultures were done given lactic acidosis as a part of infectious work-up and was noted to be negative. COVID-19 rapid polymerase chain reaction (PCR) test was done and noted to be negative. Viral hepatitis panel was checked and was negative on admission. Given the abnormal labs and inconsistency with his history of isopropyl alcohol ingestion especially given significantly elevated anion gap acidosis, consultation with a toxicologist was obtained. Upon review, the toxicologist was under the impression that the patient might have had possible mixed toxic alcohol ingestion and recommended the patient to be started on fomepizole drip with a loading dose of 50 mg/kg and to continue maintenance at 10 mg/kg every 12 hours for four doses. Also recommended to start the patient on leucovorin every six hours. The patient was also started on thiamine 500 mg IV on day 1 and 100 mg daily and 100 mg of vitamin B6 daily on admission. He had also received 4 g of IV magnesium sulfate and 2 g of IV calcium gluconate. Given significant anion gap acidosis and lactic acidosis with severe electrolyte abnormalities, the patient was admitted to ICU for the first 24 hours. He was started on the Clinical Institute Withdrawal Assessment protocol given his known history of alcohol dependency. We did send out toxic alcohol levels to an outside lab and only acetone of 71 mg/dL was noted. Ethylene glycol, isopropyl alcohol, and methanol were not detected. Given that isopropyl alcohol, ethylene glycol and methanol were undetected, omeprazole and leucovorin were promptly discontinued. The patient also had a brief episode of nonsustained ventricular tachycardia likely secondary to severe hypokalemia which was promptly corrected with a total of 120 mEq of oral potassium and 40 mEq of IV potassium. The patient also had a prolonged QTC of 586 ms on admission and improved to 503 ms within 24 hours with electrolyte repletion. The patient had improved clinically and was discharged on day 4 of admission.

## Discussion

**Figure 1:**
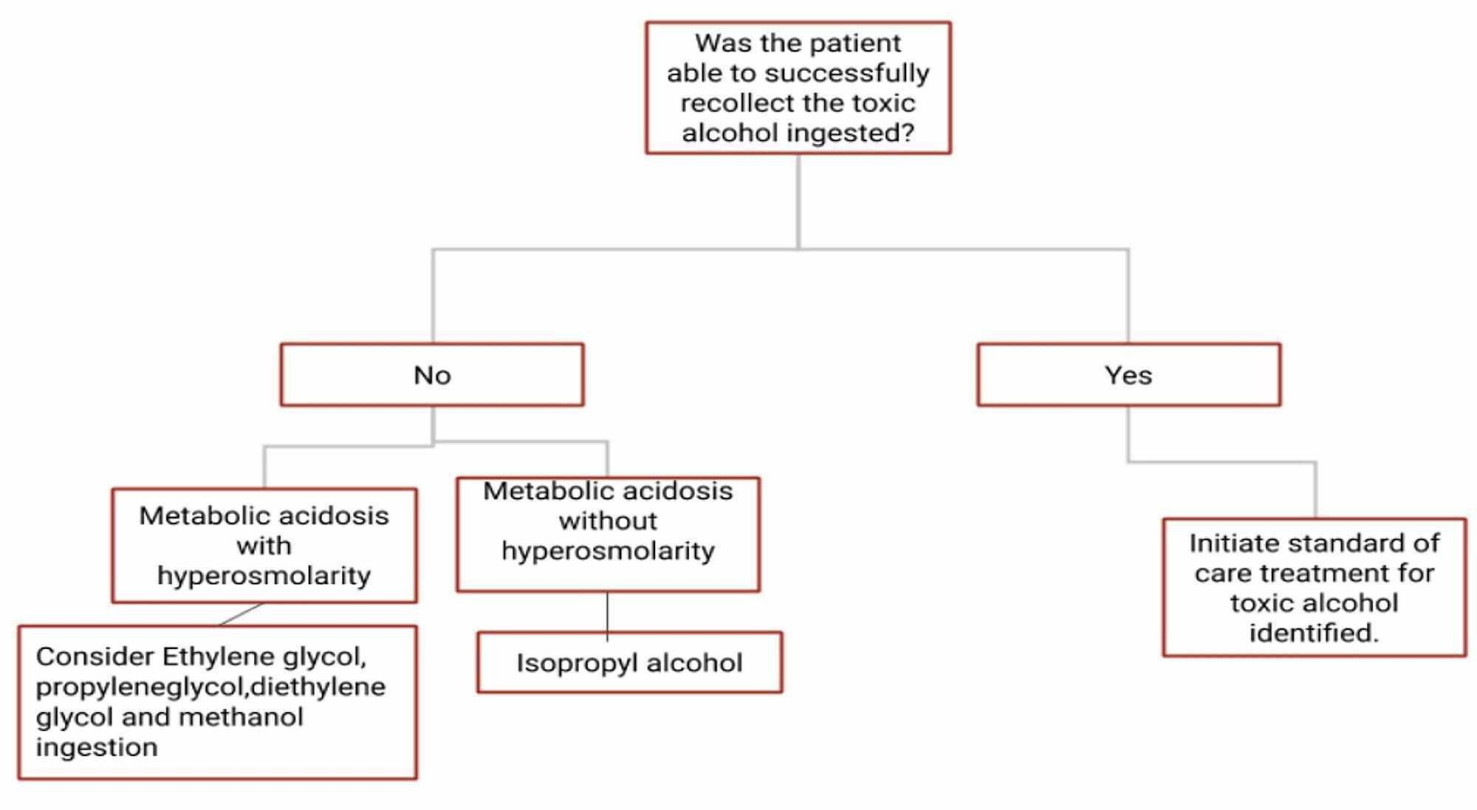
Management pathway based on history and laboratory findings.

Toxic alcohol ingestion can be fatal or produce irreversible tissue damage and hence timely recognition and treatment are very important. There are some key characteristics and clinical presentations that can help us achieve timely diagnosis aiding us in initiating appropriate treatment as soon as possible (Figure 1, Table 1). 

**Table 1:** Brief overview of various toxic alcohols, their common clinical presentations, common laboratory abnormalities, and treatment.

Type of alcohol	Toxicity causing substance(s)	Common clinical features	Common laboratory abnormalities	Treatment	Comments
Methanol	Formic acid, lactic acid, ketones	Retinal damage with blindness, rarely putaminal damage with neurological dysfunction	Metabolic acidosis with hyperosmolarity	Fomepizole with or without hemodialysis	Mortality rate can be high if not treated quickly
Ethylene glycol	Glycolic acid, calcium oxalate	Acute renal failure, myocardial and cerebral damage	Metabolic acidosis with hyperosmolarity	Fomepizole with or without hemodialysis	Most common toxic alcohol ingestion especially in children
Isopropyl alcohol	Isopropyl alcohol	Coma, hypotension	Hyperosmolarity without significant metabolic acidosis	Supportive treatment is usually sufficient, initiate hemodialysis with serum level of 200-400 mg/dL or in the presence of marked hypotension,	Positive nitroprusside reaction
Diethylene glycol	2-hydroxyethoxy acetic acid	Acute renal failure, neurological damage	Metabolic acidosis with hyperosmolarity	Hemodialysis	Associated with high mortality due to late recognition and treatment
Propylene glycol	Lactic acid	Minimal clinical abnormalities	Metabolic acidosis with hyperosmolarity	Discontinue medication containing propylene glycol is usually effective but in patients with serum concentrations greater than 400 mg/dL or severe lactic acidosis might benefit from hemodialysis	May be most common alcohol intoxication in ICU setting

Methanol intoxication can be seen in either accidental or intentional ingestion of adulterated alcohol or products with methanol and in rare cases can also be seen in inhalation of methanol. Ethylene glycol intoxication can be seen in accidental or intentional ingestion of antifreeze, alcohol adulterated with ethylene glycol, or products with ethylene glycol. Diethylene glycol intoxication is seen in ingestion of contaminated medication or products with diethylene glycol. Propylene glycol intoxication is usually seen in IV administration of medication with propylene glycol and rarely seen in ingestion of products with propylene glycol. Isopropyl alcohol intoxication can be seen in accidental or intentional ingestion of rubbing alcohol [1]. 

Diethylene glycol intoxication poses very high mortality possibly related to late recognition and treatment since it is most commonly associated with ingestion of contaminated medications or commercial products. Hyperosmolality can be less frequently seen then with other toxic alcohols. Ethylene glycol ingestion seems to be more frequently seen than methanol intoxication especially in children. The substances causing toxicity in methanol intoxication are formic acid, lactic acid, and ketones. Glycolic acid and calcium oxalate are the substances that cause toxicity in ethylene glycol intoxication. 2-hydroxythoxyacetatic acid is the substance causing toxicity in diethylene glycol intoxication. Lactic acid is the substance causing toxicity in propylene glycol intoxication. Isopropanol is the substance causing toxicity in isopropanol intoxication [1]. 

The normal serum osmolality is around 285-290 mOsm/L. Serum osmolality can be either calculated or measured by freezing point depression. Accumulation of the low molecular weight substances in the serum such as toxic alcohols will raise the measured serum osmolality producing an osmolal gap. Osmolal gap is the difference between measured and calculated serum osmolality [1,3]. The difference is usually within 10 mOsm/L. Serum osmolality can be calculated by using the following equation [1].

Serum osmolality = 2XNa + Blood urea nitrogen (BUN)/2.8 + Blood glucose/18.

Osmolal gap with high anion gap metabolic acidosis can be commonly seen in methanol, ethylene glycol, and diethylene glycol intoxications. Propylene glycol intoxication can cause osmolal gap with or without lactic acidosis. Isopropyl alcohol intoxication causes an osmolal gap without high anion gap metabolic acidosis. Anion gap varies depending on the time elapsed since ingestion of the toxic alcohol and the ongoing metabolism of the ingested toxic alcohol [2]. With time since ingestion increases, an increase in anion gap and a decrease in osmolal gap is observed [2]. Ethanol levels tend to increase the osmolal gap and it is vital to check its level as was done in our patient for accurate results [2]. Visual disturbances with optic papillitis can be seen in methanol intoxication. Osmolal gap with acute renal failure can be seen in patients with ethylene glycol and dietary glycol intoxication. Calcium oxalate crystals in urine, monohydrate or dihydrate crystals can be seen in ethylene glycol intoxication. Osmolal gap along with coma can be seen in diethylene glycol intoxication. Poor prognostic factors include blood pH with less than 7.1, severe lactic acidosis, severe hypotension, severe coma, acute renal failure requiring hemodialysis, and diagnosis greater than 10 hours after ingestion [1]. 

General principles and treatment of alcohol intoxication include gastric lavage or use of activated charcoal to remove alcohol from gastrointestinal tract but it needs to be initiated within 30-60 minutes after ingestion of alcohol. Administration of ethanol or fomepizole to delay or prevent generation of toxic metabolites needs to be initiated while sufficient alcohol remains to be metabolized and measurement of blood alcohol concentrations and/or serum osmolality can be helpful. The recommended dose of fomepizole for patients with methanol and ethylene glycol intoxication not requiring dialysis is a loading dose of 50 mg/kg body weight with maintenance dose of 10 mg/kg body weight every 12 hours for four doses or 15 mg/kg body weight every 12 hours. Dialysis is helpful in removing unmetabolized alcohol and possibly toxic metabolites and delivering base to patients to ameliorate metabolic acidosis. In patients requiring dialysis add 1-1.5 mg/kg body weight per hour to the standard dose of fomepizole. Hemodialysis can be initiated in all the toxic alcohol ingestion cases with poor prognostic factors and when serum concentration levels are readily available [1-2].

## Conclusions

Toxic alcohol ingestion is most often a challenging clinical situation for a physician. An accurate history may not always be available. Reliance on clinical features and laboratory findings is often needed by the clinician. Our case highlights the impedance a physician finds in such cases. Toxic alcohol ingestion requires time sensitive management measures. Assessment of the airway, contacting poison control, consideration of ethanol or fomepizole for blocking the toxic metabolites, correction of acidosis, replenishing of cofactors and correction of electrolyte abnormalities and assessing for the need of dialysis form the cornerstone of the management plan.
